# Engagement, resilience, and social–emotional competence in university sports dance education: a moderated mediation model

**DOI:** 10.3389/fpsyg.2026.1897483

**Published:** 2026-09-08

**Authors:** Yaqi Liu, Wei Liu, Yue Zhang

**Affiliations:** 1 Graduate School, José Rizal University, Mandaluyong City, Philippines; 2 School of Physical Education, Jiangsu Ocean University, Lianyungang, Jiangsu, China; 3 School of Music and Dance, Daqing Normal University, Daqing, Heilongjiang, China

**Keywords:** embodied learning, higher education, psychological resilience, social–emotional competence, sports dance learning engagement, supportive climate

## Abstract

**Objective:**

This study examined the associations among sports dance learning engagement, psychological resilience, perceived supportive climate, and social–emotional competence (SEC), with particular attention to a hypothesized indirect association through psychological resilience and whether the engagement–resilience association varied across levels of supportive climate.

**Methods:**

Cross-sectional self-report data were collected from 615 undergraduate students enrolled in sports dance courses at three universities in China. Latent-variable structural equation modeling was used to examine the direct and indirect associations, and PROCESS Model 7 was used as a complementary observed-score analysis of the conditional indirect association.

**Results:**

Sports dance learning engagement was positively associated with psychological resilience (*β* = 0.442, *p* < 0.001) and SEC (*β* = 0.364, *p* < 0.001), while psychological resilience was positively associated with SEC (*β* = 0.375, *p* < 0.001). A statistically significant indirect association between engagement and SEC through psychological resilience was observed (*β* = 0.166, 95% Monte Carlo CI [0.120, 0.213]). The engagement–resilience association was larger at higher levels of perceived supportive climate, and the index of moderated mediation was statistically significant (0.053, 95% CI [0.027, 0.080]).

**Conclusion:**

The findings support a statistically significant moderated indirect-association model in cross-sectional self-report data. They are consistent with the theoretically specified model but do not establish temporal sequencing, causal effects, or a developmental mechanism.

## Introduction

Social–emotional competence (SEC) has emerged as an important outcome in higher education because it is associated with students’ emotional regulation, interpersonal functioning, psychological adjustment, and responsible decision-making (Wang et al., 2025; Lin and Wang, 2025). As universities place greater emphasis on student well-being and holistic development, increasing attention has been directed toward educational experiences that support psychosocial functioning alongside academic achievement (Xuan, 2025). Physical education and structured physical activity provide one relevant educational context because participation involves not only physical and motor demands but also psychological and social experiences. Research has highlighted the broader relevance of physical education and structured physical activity to students’ physical, mental, and social functioning (Sortwell et al., 2025). Individual characteristics, basic psychological needs, and enjoyment have also been associated with the quality and persistence of exercise participation (Teixeira et al., 2021). Within this broader context, embodied learning activities have attracted growing interest because they integrate cognitive, emotional, physical, and social dimensions of learning (Hayashi et al., 2022).

Sports dance provides a relevant setting for examining these relationships because it combines physical movement, artistic expression, interpersonal interaction, rhythm, feedback, and socially visible performance. Dance-based learning has been discussed in relation to embodied cognition, nonverbal expression, interpersonal synchrony, and supportive learning environments (Borowski, 2023; Zhang and Wei, 2024). In higher education, dance-related and physical activity-based experiences have also been associated with interpersonal competence, emotional regulation, psychological well-being, and social adaptability (Ni and Chen, 2025; Yang and Li, 2022; Li et al., 2024). Students in sports dance may be required to communicate through movement, coordinate with partners, respond to corrective feedback, and perform in socially evaluative situations. Dance-based learning research has reported associations with emotional expression, social connectedness, interpersonal communication, and psychosocial well-being (Domene and Morley, 2022; Delattre et al., 2024; Zafeiroudi, 2023). Related research on embodied movement practices has also linked bodily engagement with emotional awareness, social relationships, and psychological well-being (Shim et al., 2024; Zafeiroudi et al., 2025). However, the present study does not assume that sports dance is superior to, or fundamentally distinct from, other forms of physical activity, dance, arts-based education, or collaborative learning. Rather, it is treated as a theoretically relevant educational context in which these psychological and social relationships can be examined.

Learning engagement represents one potentially relevant correlate of social–emotional functioning in this context. Engagement refers to students’ behavioral, emotional, and cognitive involvement in learning activities (Fredricks et al., 2004). Previous research has consistently demonstrated positive associations between engagement and academic achievement, motivation, persistence, and well-being (Ledertoug and Paarup, 2021; Zhang, 2022). Recent higher education studies have also linked engagement with emotional intelligence, self-efficacy, motivation, and social–emotional functioning (Chang and Tsai, 2022; Elmi, 2020). Psychological resilience may be particularly relevant to this association because it refers to the capacity to adapt positively and recover from challenge, stress, and adversity (Connor and Davidson, 2003; Masten, 2001). In sports dance, performance pressure, repeated correction, physical fatigue, partner-coordination demands, and public evaluation make adaptive functioning in response to setbacks theoretically salient. Broaden-and-Build Theory provides a theoretical rationale for specifying a positive association between engagement and adaptive psychological resources such as resilience (Fredrickson, 2001). However, because the present study is cross-sectional, any proposed ordering among engagement, resilience, and SEC is theoretical rather than temporally demonstrated.

The learning environment may also be relevant to the engagement–resilience association. Conservation of Resources Theory suggests that supportive environments provide contextual resources that may facilitate the maintenance and use of personal resources (Hobfoll, 1989). Research in higher education has associated supportive teacher–student relationships, peer support, and positive learning environments with resilience, psychological well-being, and adaptive functioning (Lin and Wang, 2025; Zhu and Ahmad, 2026). Related work has also emphasized the relevance of supportive educational contexts to students’ psychological adjustment and adaptive functioning (Marrone et al., 2024). In sports dance settings, teacher encouragement, peer support, and psychological safety may be especially relevant when students encounter mistakes, feedback, interpersonal coordination demands, and performance pressure. Accordingly, supportive climate is examined here as a contextual condition associated with variation in the engagement–resilience relationship rather than as a demonstrated cause of resilience development. The specific placement of supportive climate on the engagement–resilience association is developed further in the hypothesis section, where its theoretical proximity to challenge-management experiences is considered.

Existing research has therefore established relevant associations involving engagement, resilience, supportive educational environments, dance or physical-activity participation, and psychosocial functioning, but these lines of research have largely developed separately. The empirical gap addressed in the present study concerns the specific configuration of these constructs within university sports dance education: whether sports dance learning engagement is associated with SEC indirectly through psychological resilience and whether the engagement–resilience association varies according to students’ perceptions of supportive climate. The study does not propose that sports dance requires a separate theoretical or developmental model; rather, its value lies in testing whether established educational-psychology relationships are observable within a movement-based, partner-coordinated, and socially evaluative learning context. Because all focal constructs were assessed concurrently, the model is interpreted as a theory-informed pattern of direct, indirect, and conditional associations rather than as evidence of temporal sequencing or a developmental mechanism. Its contribution is therefore primarily contextual and integrative, providing a context-specific test of established relationships among engagement, adaptive psychological functioning, perceived environmental support, and SEC.

### Theoretical framework and hypotheses development

#### Sports dance learning engagement and social–emotional competence

Social–emotional competence (SEC) broadly refers to students’ capacities to understand and regulate emotions, maintain constructive relationships, and make responsible decisions in social and learning contexts (Gimbert et al., 2023). The CASEL framework conceptualizes social–emotional competence across multiple interrelated domains (Collaborative for Academic, Social, and Emotional Learning (CASEL), 2020; Durlak et al., 2011). In the present analytic model, SEC is operationalized through four CASEL-aligned dimensions: self-awareness, self-management, relationship skills, and responsible decision-making (Carpendale et al., 2023). This four-dimensional specification therefore represents the operational definition used in the present study rather than an exhaustive representation of every domain within the broader CASEL framework. In higher education, these competencies are relevant to students’ psychological adjustment, interpersonal functioning, and adaptive participation in university life (Wang et al., 2025).

Sports dance provides a theoretically relevant setting in which to examine these competencies because it combines physical movement, emotional expression, artistic performance, and interpersonal coordination. Students may be required to communicate through movement, synchronize actions with partners, respond to rhythm and feedback, and perform in socially evaluative situations. Dance experiences have been associated with communication, social interaction, and embodied expression (Nesbit et al., 2025; Ozawa-de Silva and Ravi, 2026). These characteristics provide a plausible context in which emotion regulation, self-awareness, cooperation, and responsible interaction are relevant to students’ learning experiences. However, because the present study does not compare sports dance with other educational activities, these features are treated as contextual characteristics rather than evidence that sports dance has uniquely stronger social–emotional effects.

Learning engagement reflects students’ behavioral, emotional, and cognitive involvement in educational activities (Fredricks et al., 2004). In sports dance, students reporting greater engagement may also report stronger behavioral participation, emotional involvement, and cognitive investment in learning and performance improvement. From a social–emotional learning perspective, such involvement occurs within structured, interactive, and emotionally meaningful experiences that are theoretically relevant to social–emotional functioning (Collaborative for Academic, Social, and Emotional Learning (CASEL), 2020; Durlak et al., 2011; Czauderna et al., 2024). Accordingly, greater sports dance learning engagement is expected to be positively associated with greater SEC. This expectation concerns a contemporaneous association and does not imply that engagement causes subsequent development of SEC.

*H1*: Sports dance learning engagement is positively associated with social-emotional competence.

### Psychological resilience as an indirect link between engagement and social–emotional competence

Psychological resilience refers to students’ capacity to adapt positively and recover from setbacks when facing difficulty or stress (Connor and Davidson, 2003; Masten, 2001). It is theoretically relevant in sports dance learning because students may encounter repeated practice demands, performance evaluation, partner-coordination challenges, physical fatigue, and public demonstration pressure (Li et al., 2025). These conditions make adaptive responses to mistakes, pressure, and setbacks particularly salient. Resilience is therefore positioned as a theoretically relevant intermediate construct because it is related to, but conceptually distinguishable from, both learning engagement and SEC. Engagement concerns students’ behavioral, emotional, and cognitive involvement in learning, whereas resilience primarily concerns adaptive functioning in response to difficulty, stress, and setbacks. Compared with self-efficacy, which primarily reflects confidence in performing a task successfully, resilience more directly concerns recovery and adaptation when challenges occur. Previous research has also associated learning involvement and supportive educational experiences with adaptive psychological functioning (Ledertoug and Paarup, 2021; Marrone et al., 2024; Wang et al., 2025).

Resilience and SEC nevertheless share some conceptual proximity, particularly in relation to emotion regulation and adaptive responding. Resilience primarily concerns the capacity to recover from and adapt to setbacks and adversity (Connor and Davidson, 2003; Masten, 2001), whereas SEC, as operationalized in the present study, encompasses a broader set of competencies involving self-awareness, self-management, relationship skills, and responsible decision-making (Collaborative for Academic, Social, and Emotional Learning (CASEL), 2020). Self-management is therefore the SEC dimension most conceptually adjacent to resilience, but the two constructs are not equivalent: resilience is defined around adaptation to challenge and adversity, whereas self-management concerns broader regulation of emotions and behavior across learning and social situations. Their empirical distinctiveness is therefore evaluated through the measurement model and discriminant-validity analyses rather than assumed solely on theoretical grounds.

Broaden-and-Build Theory provides a theoretical rationale for specifying a positive association between engagement and psychological resilience. The theory proposes that positive emotional experiences can broaden individuals’ momentary thought-action repertoires and, over time, contribute to enduring psychological resources (Fredrickson, 2001). Engagement itself is not equivalent to the positive emotions specified in Broaden-and-Build Theory; rather, students who report greater engagement in sports dance may also report accomplishment, enjoyment, social connectedness, and mastery through practice. Such engagement-related experiences have been discussed in relation to adaptive psychological functioning in dance and movement contexts (Borowski, 2023; Zafeiroudi, 2023; Delattre et al., 2024). In the present cross-sectional study, the theory therefore provides a broader rationale for the observed engagement–resilience association rather than evidence that engagement preceded or produced the development of resilience.

*H2*: Sports dance learning engagement is positively associated with psychological resilience.

Psychological resilience is also expected to be positively associated with SEC. Students reporting greater resilience may also report greater capacity to regulate emotional responses, manage stress, maintain constructive relationships, and make responsible decisions when facing challenging situations. Within sports dance learning, these capacities may be reflected in responding adaptively to mistakes, sustaining cooperation with partners, accepting feedback, and maintaining self-regulation in socially evaluative performance settings. Taken together, the theoretical model specifies an indirect statistical association in which sports dance learning engagement is positively associated with psychological resilience, which in turn is positively associated with SEC. Because the three constructs were assessed concurrently, this ordering is theoretically specified rather than temporally demonstrated. A statistically significant indirect effect would therefore indicate that the observed covariance structure is consistent with the proposed theoretical ordering, rather than demonstrating that engagement precedes resilience or that resilience subsequently produces changes in SEC.

*H3*: Psychological resilience is positively associated with social-emotional competence.

*H4*: Sports dance learning engagement is positively associated with social-emotional competence indirectly through psychological resilience.

### Supportive climate as a contextual moderator

Supportive climate refers to students’ perceptions that the learning environment provides encouragement, assistance, acceptance, and psychological safety (Edmondson, 1999; Robinson and Held, 2025). In sports dance education, these contextual characteristics may be particularly relevant because students perform in front of others, cooperate with partners, and receive feedback on visible bodily performance. Supportive learning environments have been associated with students’ willingness to remain involved in challenging learning activities (Prananto et al., 2025), while learning-focused and supportive classroom climates in physical education have also been associated with engagement and motivation (Marcelino, 2025).

Conservation of Resources Theory provides the theoretical rationale for examining supportive climate as a contextual moderator. The theory proposes that individuals seek to acquire, preserve, and protect valued resources and that environmental resources may be relevant to the availability and maintenance of personal resources (Hobfoll, 1989). Within sports dance learning, teacher support, peer encouragement, and psychological safety can be conceptualized as contextual resources because they provide emotional reassurance, instructional guidance, interpersonal assistance, and reduced concern about negative interpersonal consequences (Gao et al., 2025; Prananto et al., 2025). These resources may be particularly relevant when students respond to correction, mistakes, interpersonal coordination demands, and performance pressure (Robinson and Held, 2025).

Supportive climate is hypothesized to moderate specifically the engagement-resilience association because this path concerns the relationship between students’ active learning involvement and their adaptive functioning in the face of challenge. Engagement may be associated with greater exposure to practice, feedback, performance demands, and opportunities for mastery, whereas resilience concerns adaptive responses when such experiences involve difficulty or setbacks. Teacher encouragement, peer cooperation, and psychological safety are therefore theoretically proximal to the circumstances under which engagement and adaptive responses to challenge co-occur. This rationale is more direct for the engagement-resilience association than for the resilience-SEC association, which concerns the relationship between an individual adaptive capacity and broader self-regulatory and interpersonal competencies. Accordingly, the engagement-resilience association is expected to be stronger at higher levels of perceived supportive climate. This expectation is theory-informed rather than evidence that supportive climate causally changes the effect of engagement on resilience; the present cross-sectional analysis tests whether the statistical association differs across levels of perceived supportive climate.

*H5*: Supportive climate moderates the association between sports dance learning engagement and psychological resilience, such that the positive association is stronger at higher levels of supportive climate.

**Figure 1:**
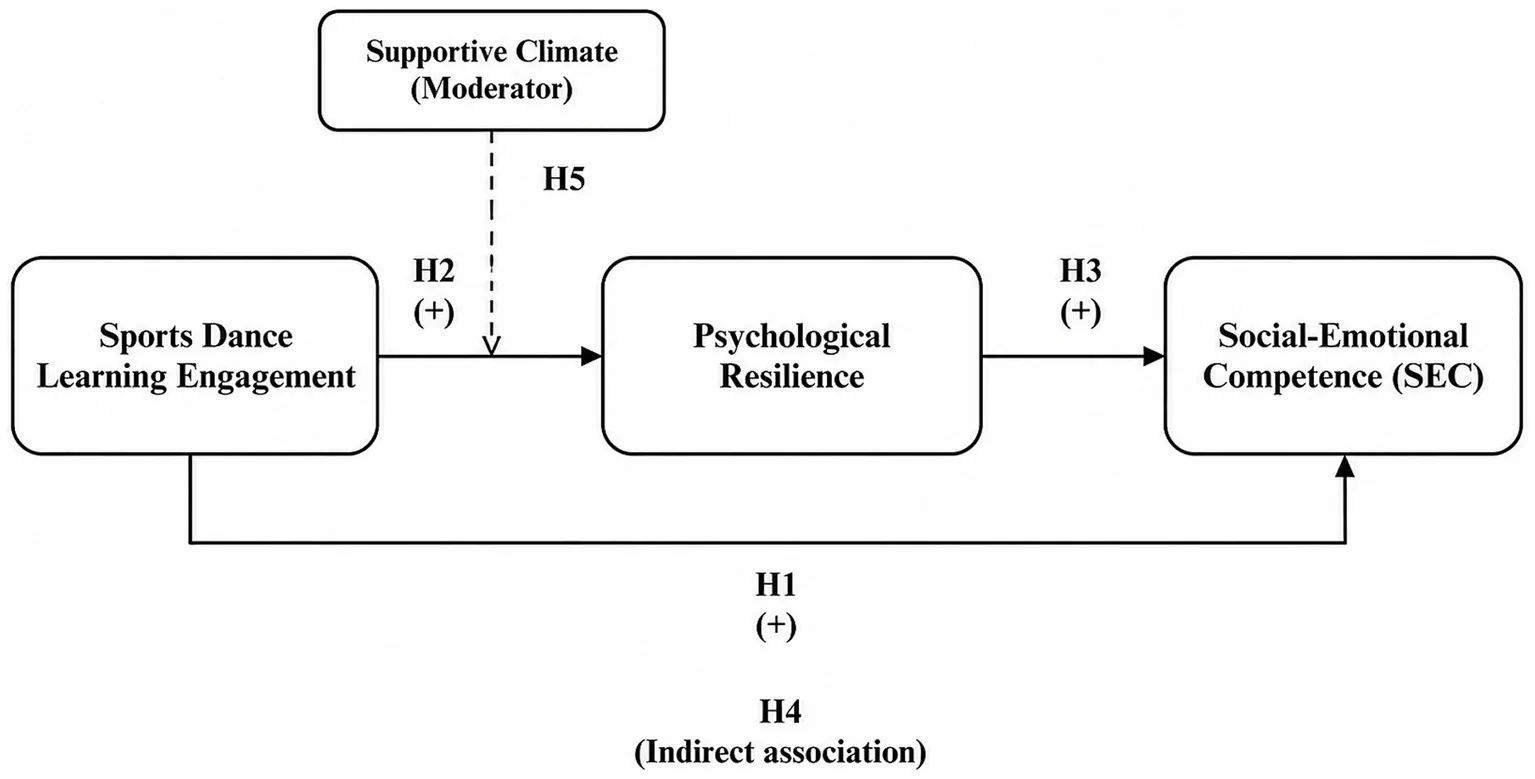
Proposed model of sports dance learning engagement, psychological resilience, supportive climate, and social–emotional competence. Arrows represent theoretically specified statistical associations. Psychological resilience is modeled as an intermediate variable in the indirect association between sports dance learning engagement and social–emotional competence, whereas supportive climate is modeled as a moderator of the engagement-resilience association. Because all constructs were measured cross-sectionally, the figure does not establish temporal or causal ordering.

Based on the above hypotheses, the present study evaluates the model shown in Figure 1. Sports dance learning engagement is specified as being directly associated with SEC and indirectly associated with SEC through psychological resilience, while supportive climate is specified as a first-stage moderator of the engagement-resilience association. Because all focal constructs were assessed concurrently, the directional paths represent theoretically specified statistical associations rather than established temporal or causal relationships.

## Methods

### Research design

This study employed a quantitative cross-sectional survey design to examine the associations among sports dance learning engagement, psychological resilience, supportive climate, and social–emotional competence (SEC) among Chinese university students. Guided by Social–Emotional Learning perspectives, Broaden-and-Build Theory, and Conservation of Resources Theory, the study evaluated a theory-informed conditional indirect-association model in which psychological resilience was specified as an intermediate variable in the association between sports dance learning engagement and SEC, while supportive climate was specified as a moderator of the engagement–resilience association. Because all focal variables were assessed concurrently, the directional specification of the model reflects the proposed theoretical ordering and does not establish temporal or causal relationships.

### Participants and sampling procedure

Participants were undergraduate students enrolled in sports dance courses at three universities in China during the 2025–2026 academic year. Eligibility required completion of at least one semester of sports dance learning. Following permission from the relevant university personnel and course instructors, eligible students were invited to complete an anonymous online questionnaire. Participation was voluntary, and electronic informed consent was obtained before questionnaire completion. The study therefore represents a course-based sample of students with prior exposure to university sports dance instruction rather than a probability sample of the broader Chinese university student population.

A total of 648 questionnaires were returned. Responses showing patterned responding or completion times shorter than 3 min were excluded during data screening. After data screening, 615 responses were retained for analysis, representing 94.91% of the returned questionnaires. The final sample included 261 male students (42.4%) and 354 female students (57.6%) and represented all four undergraduate years. Participants also varied in sports dance experience and weekly practice frequency, as summarized in Table 1.

**Table 1:** Demographic characteristics of participants (*N* = 615).

Variable	Category	*n*	%
Gender	Male	261	42.4
	Female	354	57.6
Academic year	First year	177	28.8
	Second year	142	23.1
	Third year	156	25.4
	Fourth year	140	22.8
Sports dance experience	<1 year	184	29.9
	1–2 years	201	32.7
	2–3 years	129	21
	>3 years	101	16.4
Weekly practice frequency	Once	126	20.5
	Twice	200	32.5
	Three times	195	31.7
	Four or more times	94	15.3
Practice format	Mainly solo practice	155	25.2
	Mainly partner-based practice	316	51.4
	Both solo and partner-based practice	144	23.4

Class- and institution-level identifiers were not retained in the final analytic dataset; therefore, class-specific sample sizes, ICCs, and potential clustering effects could not be estimated retrospectively, and the analyses were conducted at the individual level. Practice format and weekly practice frequency are reported in Table 1, whereas course status and competitive versus recreational participation were not assessed.

### Measures

All focal constructs were assessed using five-point Likert-type items ranging from 1 (strongly disagree) to 5 (strongly agree), with higher scores indicating higher levels of the corresponding construct. The measurement structure comprised 12 items for sports dance learning engagement, six items for psychological resilience, 16 items for social–emotional competence (SEC), and 12 items for supportive climate. The measures were derived from established theoretical frameworks and previously developed measures and were adapted, where necessary, to the university sports dance context while retaining the intended construct content and dimensional structure. Because the measures were used in contextually adapted forms, their psychometric properties were evaluated in the present sample before testing the hypothesized structural relationships.

### Sports dance learning engagement

Sports dance learning engagement was assessed using 12 contextually adapted items representing three established dimensions of student engagement: behavioral engagement, emotional engagement, and cognitive engagement, with four items for each dimension. The dimensional structure was based on the multidimensional engagement framework of Fredricks et al. (2004). Behavioral engagement reflected active participation and effort in sports dance learning, emotional engagement reflected students’ affective involvement in learning activities, and cognitive engagement reflected thoughtful and strategic investment in learning and performance improvement. Item wording was adapted to refer specifically to university sports dance learning while retaining the intended content of the corresponding engagement dimension. A sample item was, “I actively participate in sports dance learning activities.” The three dimensions were modeled as indicators of an overall sports dance learning engagement construct.

### Psychological resilience

Psychological resilience was assessed using six contextually adapted items reflecting students’ perceived capacity to recover from difficulties, adapt to setbacks, and maintain adaptive functioning when encountering challenges in sports dance learning and performance. The measure drew on established resilience measurement traditions, with item wording contextualized to the sports dance learning setting while retaining the intended meaning of psychological adaptation and recovery. A sample item was, “I can quickly recover after experiencing difficulties in sports dance practice.” The six items were modeled as indicators of a single psychological resilience construct.

### Social–emotional competence

Social–emotional competence was assessed using 16 contextually adapted items representing four dimensions: self-awareness, self-management, relationship skills, and responsible decision-making, with four items for each dimension. The dimensional organization was aligned with corresponding domains of the CASEL framework (Collaborative for Academic, Social, and Emotional Learning (CASEL), 2020). Self-awareness reflected students’ awareness of their emotions and personal responses, self-management reflected regulation of emotions and behavior, relationship skills reflected constructive interpersonal functioning, and responsible decision-making reflected considered and responsible choices in learning and social situations. Item wording was contextualized to university learning and sports dance-related situations while preserving the intended content of the corresponding social–emotional domain. A sample item was, “I can regulate my emotions when facing learning challenges.” The four dimensions were modeled as indicators of an overall SEC construct.

### Supportive climate

Supportive climate was assessed using 12 contextually adapted items representing three established aspects of supportive learning environments: teacher support, peer support, and psychological safety, with four items for each dimension. Teacher support reflected perceived encouragement and instructional assistance from instructors, peer support reflected interpersonal assistance and encouragement from classmates, and psychological safety reflected students’ perceptions that they could participate, make mistakes, and express themselves without undue interpersonal concern. The psychological-safety dimension was informed by established work on psychological safety, including Edmondson (1999), while the teacher- and peer-support items reflected established educational support constructs. Item wording was adapted to the university sports dance context. A sample item was, “My instructor encourages me when I encounter difficulties.” The three dimensions were modeled as indicators of an overall supportive-climate construct.

Measures originally developed or described in English were translated into Chinese and subsequently back-translated to examine semantic correspondence. Where contextual adaptation was required, changes were limited to references to university sports dance learning and did not intentionally alter the underlying construct content. Three experts with backgrounds in educational psychology, physical education, and sports dance reviewed the translated and adapted items for conceptual correspondence, clarity, and contextual appropriateness. A pilot test involving 50 university students was conducted before formal data collection to assess item comprehensibility and preliminary internal consistency. The pilot results indicated satisfactory item clarity, with Cronbach’s alpha coefficients above 0.80 for the focal measures.

### Assessment of potential common method variance

Because all focal variables were assessed using self-report questionnaires at the same measurement occasion, procedural and statistical approaches were used to evaluate potential common method variance. Procedurally, anonymity and confidentiality were emphasized, and participants were informed that there were no right or wrong answers. Statistically, Harman’s single-factor test and full-collinearity variance inflation factors were examined as preliminary diagnostics. In addition, confirmatory factor analytic model comparisons were used to examine whether the covariance among the measurement items could be adequately represented by a substantially more restrictive single-factor specification. Because no single statistical procedure can eliminate the possibility of common method variance, common method variance was treated as a potential source of bias rather than considered definitively absent.

### Data analysis procedure

Data were analyzed using SPSS 29.0, AMOS 26.0, and Hayes’ PROCESS Macro Version 4.2. Preliminary analyses included data screening, descriptive statistics, internal-consistency estimates, and Pearson correlations among the focal variables. Confirmatory factor analysis (CFA) was then conducted to evaluate the measurement structure before testing the hypothesized structural relationships. The measurement model specified sports dance learning engagement as a higher-order construct represented by behavioral, emotional, and cognitive engagement; social–emotional competence (SEC) as a higher-order construct represented by self-awareness, self-management, relationship skills, and responsible decision-making; and supportive climate as a higher-order construct represented by teacher support, peer support, and psychological safety. Psychological resilience was modeled as a single latent construct. Internal consistency was evaluated using Cronbach’s alpha and composite reliability, convergent validity using average variance extracted (AVE), and discriminant validity using heterotrait–monotrait ratios (HTMT). Model fit was evaluated using chi-square (χ^2^), χ^2^/df, CFI, TLI, RMSEA, and SRMR.

Latent-variable structural equation modeling constituted the primary inferential analysis. The structural model was used to estimate the hypothesized associations among sports dance learning engagement, psychological resilience, and SEC. Model-based standard errors and 95% confidence intervals were reported for the individual structural paths. The indirect association between sports dance learning engagement and SEC through psychological resilience was evaluated using 5,000 Monte Carlo draws based on the estimated parameter covariance matrix. An indirect association was considered statistically distinguishable from zero when its 95% Monte Carlo confidence interval excluded zero.

PROCESS Model 7 was used as a complementary observed-score analysis to estimate the first-stage interaction involving supportive climate and to probe the conditional indirect association at different levels of supportive climate. For this analysis, observed composite scores were calculated by averaging the items corresponding to each focal construct, and the four composite variables were standardized before being entered into the PROCESS model. Sports dance learning engagement was specified as the predictor, psychological resilience as the intermediate variable, supportive climate as the first-stage moderator, and SEC as the outcome. The interaction was probed at lower (−1 SD), mean, and higher (+1 SD) levels of supportive climate. Model-based confidence intervals were reported for the regression coefficients and simple slopes, whereas the conditional indirect associations and the index of moderated mediation were evaluated using 5,000 bootstrap resamples and bootstrap 95% confidence intervals. PROCESS results were interpreted as complementary to, rather than a replacement for, the latent-variable SEM because PROCESS operates on observed composite scores. A unified latent moderated mediation model with latent interaction terms was not estimated because the prespecified analytical strategy separated the primary latent-variable assessment of the measurement and structural associations from the complementary probing of the interaction and conditional indirect pattern. Latent-variable SEM was therefore retained as the primary inferential framework for the direct and indirect associations, whereas the observed-score conditional process analysis was used specifically to obtain readily interpretable interaction estimates, simple slopes, conditional indirect associations, and the index of moderated mediation. Because the latter analysis was based on observed composite scores and did not model measurement error, its moderation results were interpreted as complementary and were not treated as equivalent to estimates from a fully latent moderated mediation model. Future research could evaluate the entire conditional indirect model within a latent-interaction framework to examine the robustness of the present observed-score moderation results. Given the cross-sectional design, neither the indirect-effect analysis nor the moderated indirect-effect analysis was interpreted as establishing temporal mediation or causal moderation; instead, the analyses evaluated whether the observed pattern of associations was consistent with the theoretically specified conditional indirect-association model.

### Ethical considerations

This study involved human participants who completed an anonymous, non-interventional educational survey. Under the institutional and national provisions applicable to anonymous, minimal-risk educational survey research, formal ethics committee review was not required for this study. No experimental intervention was administered, and no personally identifiable information was collected. Participation was voluntary, and electronic informed consent was obtained from all participants before questionnaire completion. Before providing consent, participants received information concerning the purpose of the study, the voluntary nature of participation, confidentiality of responses, and their right to discontinue participation without penalty. These procedures were established before data collection commenced on 29 December 2025.

## Results

### Common method bias

Harman’s single-factor test indicated that the first unrotated factor accounted for 25.7% of the total variance, below the recommended 40% threshold. All full-collinearity variance inflation factors were below 3.30. Furthermore, a CFA-based single-factor model was compared with the hypothesized multidimensional measurement model. The single-factor model showed substantially poorer fit to the data (χ^2^/df = 5.21, CFI = 0.712, TLI = 0.698, RMSEA = 0.113, SRMR = 0.098) than the hypothesized multidimensional model (χ^2^/df = 2.06, CFI = 0.937, TLI = 0.930, RMSEA = 0.042, SRMR = 0.035). The substantially poorer fit of the single-factor model did not support a single common-factor explanation of the observed covariance structure. However, these diagnostics cannot exclude other forms of common method variance, which therefore remained a potential limitation.

### Descriptive statistics and correlations

Table 2 presents the descriptive statistics and correlations among the main study variables. Sports dance learning engagement was positively correlated with psychological resilience (*r* = 0.378, *p* < 0.001), SEC (*r* = 0.431, *p* < 0.001), and supportive climate (*r* = 0.243, *p* < 0.001). Psychological resilience was positively correlated with SEC (*r* = 0.465, *p* < 0.001) and supportive climate (*r* = 0.172, *p* < 0.001). Supportive climate was also positively associated with SEC (*r* = 0.198, *p* < 0.001). These correlations were consistent with the hypothesized relationships.

**Table 2:** Descriptive statistics and correlations among study variables.

Variable	M	SD	1	2	3	4
1. Sports dance learning engagement	3.450	0.668	—			
2. Psychological resilience	3.460	0.736	0.378***	—		
3. Social–emotional competence	3.502	0.598	0.431***	0.465***	—	
4. Supportive climate	3.494	0.652	0.243***	0.172***	0.198***	—

*N* = 615. ***p < 0.001.

### Measurement model

Reliability and convergent validity were examined before testing the structural model. As shown in Table 3, Cronbach’s alpha coefficients ranged from 0.836 to 0.888, composite reliability values ranged from 0.836 to 0.888, and AVE values ranged from 0.568 to 0.630. Standardized factor loadings for the retained items on their respective first-order factors ranged from 0.719 to 0.825. Collectively, these results supported acceptable internal consistency and convergent validity.

**Table 3:** Reliability, convergent validity, and standardized factor loadings of the first-order dimensions.

First-order dimension	Items	M	SD	Standardized loading range	α	CR	AVE
Behavioral engagement	4	3.420	0.771	0.744–0.773	0.840	0.840	0.568
Emotional engagement	4	3.550	0.786	0.750–0.825	0.870	0.871	0.628
Cognitive engagement	4	3.381	0.792	0.756–0.802	0.853	0.853	0.593
Psychological resilience	6	3.460	0.736	0.734–0.784	0.888	0.888	0.569
Self-awareness	4	3.510	0.654	0.770–0.810	0.836	0.836	0.630
Self-management	4	3.437	0.774	0.752–0.797	0.850	0.851	0.588
Relationship skills	4	3.570	0.754	0.719–0.785	0.840	0.841	0.570
Responsible decision-making	4	3.492	0.769	0.757–0.772	0.849	0.849	0.584
Teacher support	4	3.610	0.779	0.763–0.794	0.861	0.861	0.607
Peer support	4	3.471	0.762	0.721–0.812	0.840	0.841	0.570
Psychological safety	4	3.400	0.778	0.759–0.773	0.850	0.850	0.587

Standardized loading ranges are reported for the retained indicators in the final measurement model. α = Cronbach’s alpha; CR = composite reliability; AVE = average variance extracted.

The hypothesized second-order measurement model demonstrated acceptable fit to the data, χ^2^/df = 2.06, CFI = 0.937, TLI = 0.930, RMSEA = 0.042, and SRMR = 0.035. Discriminant validity was assessed using the heterotrait–monotrait (HTMT) ratio of correlations. As shown in Table 4, HTMT values among the first-order measurement dimensions ranged from 0.117 to 0.728, and all values were below the conservative 0.85 criterion. These results supported the empirical distinctiveness of the measurement dimensions.

**Table 4:** Heterotrait–monotrait ratios among the first-order measurement dimensions.

Construct	1	2	3	4	5	6	7	8	9	10	11
1. BE	—										
2. EE	0.710	—									
3. CE	0.661	0.708	—								
4. PR	0.379	0.387	0.346	—							
5. SA	0.357	0.391	0.346	0.413	—						
6. SM	0.323	0.323	0.347	0.434	0.632	—					
7. RS	0.385	0.433	0.380	0.488	0.693	0.728	—				
8. RD	0.341	0.330	0.334	0.405	0.606	0.636	0.678	—			
9. TS	0.242	0.232	0.286	0.170	0.155	0.193	0.145	0.162	—		
10. PS	0.117	0.182	0.196	0.155	0.122	0.182	0.177	0.175	0.695	—	
11. PSY	0.167	0.170	0.247	0.176	0.173	0.171	0.129	0.184	0.657	0.647	—

BE = behavioral engagement; EE = emotional engagement; CE = cognitive engagement; PR = psychological resilience; SA = self-awareness; SM = self-management; RS = relationship skills; RD = responsible decision-making; TS = teacher support; PS = peer support; PSY = psychological safety. HTMT = heterotrait–monotrait ratio. All HTMT values were below 0.85.

### Structural model and hypothesis testing

**Figure 2:**
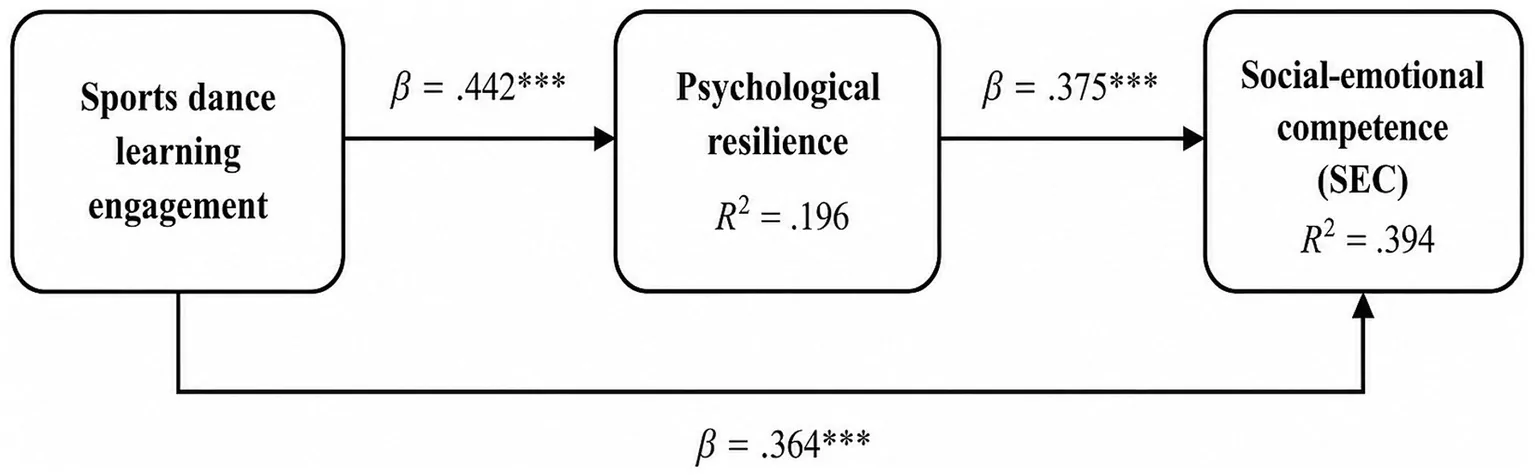
Structural equation model with standardized path coefficients for the direct and indirect associations. The moderating role of supportive climate was examined separately using PROCESS Model 7 and is presented in Figure 3 and Table 7. Path coefficients are standardized.

Following confirmation of the measurement model, the structural model was evaluated to examine the hypothesized associations among sports dance learning engagement, psychological resilience, and SEC. The structural model showed acceptable fit to the data, χ^2^(517) = 1125.46, χ^2^/df = 2.18, CFI = 0.945, TLI = 0.940, RMSEA = 0.044, and SRMR = 0.036. These indices supported an acceptable fit of the specified structural model. Figure 2 presents the latent-variable structural model for the direct and indirect associations.

As shown in Table 5, sports dance learning engagement was positively associated with SEC (*β* = 0.364, SE = 0.045, 95% CI [0.276, 0.453], *p* < 0.001), supporting H1. Engagement was also positively associated with psychological resilience (*β* = 0.442, SE = 0.040, 95% CI [0.364, 0.520], *p* < 0.001), supporting H2. Psychological resilience was positively associated with SEC (*β* = 0.375, SE = 0.043, 95% CI [0.290, 0.460], *p* < 0.001), supporting H3. Thus, students reporting greater sports dance learning engagement also tended to report greater psychological resilience and SEC, while greater psychological resilience was associated with greater SEC.

**Table 5:** Structural path results with model-based 95% confidence intervals.

Hypothesis	Path	β	SE	95% CI	*p*	Result
H1	Engagement → SEC	0.364	0.045	[0.276, 0.453]	<0.001	Supported
H2	Engagement → Psychological resilience	0.442	0.040	[0.364, 0.520]	<0.001	Supported
H3	Psychological resilience → SEC	0.375	0.043	[0.290, 0.460]	<0.001	Supported

The structural model accounted for 19.6% of the variance in psychological resilience (*R*^2^ = 0.196) and 39.4% of the variance in SEC (*R*^2^ = 0.394). These values indicate that the specified predictors accounted for a meaningful proportion of variability in the endogenous constructs.

**Table 6:** Monte Carlo analysis of the indirect association.

Effect	β	SE	95% CI	Result
Direct association: Engagement → SEC	0.364	0.045	[0.276, 0.453]	Statistically significant
Indirect association: Engagement → Psychological resilience → SEC	0.166	0.024	[0.120, 0.213]	Statistically supported

The 95% confidence interval for the indirect association was obtained from 5,000 Monte Carlo draws based on the estimated parameter covariance matrix.

### Indirect association analysis

As shown in Table 6, the indirect association between sports dance learning engagement and SEC through psychological resilience was statistically distinguishable from zero (*β* = 0.166, SE = 0.024, 95% Monte Carlo CI [0.120, 0.213]), supporting H4. The direct association between engagement and SEC also remained statistically significant (*β* = 0.364, SE = 0.045, 95% CI [0.276, 0.453]). Accordingly, the results supported a statistically significant indirect association through psychological resilience alongside a statistically significant direct association.

### Moderation and conditional indirect association

The moderating role of supportive climate was examined to determine whether the association between sports dance learning engagement and psychological resilience varied across different levels of perceived supportive climate. As shown in Table 7, the interaction between sports dance learning engagement and supportive climate was statistically significant (b = 0.150, SE = 0.036, 95% CI [0.079, 0.221], *p* < 0.001), supporting H5. The positive association between engagement and resilience was stronger at higher levels of supportive climate.

**Table 7:** Moderation result with 95% confidence interval.

Hypothesis	Path	b	SE	95% CI	*p*	Result
H5	Engagement × Supportive climate → Psychological resilience	0.150	0.036	[0.079, 0.221]	<0.001	Supported

**Figure 3:**
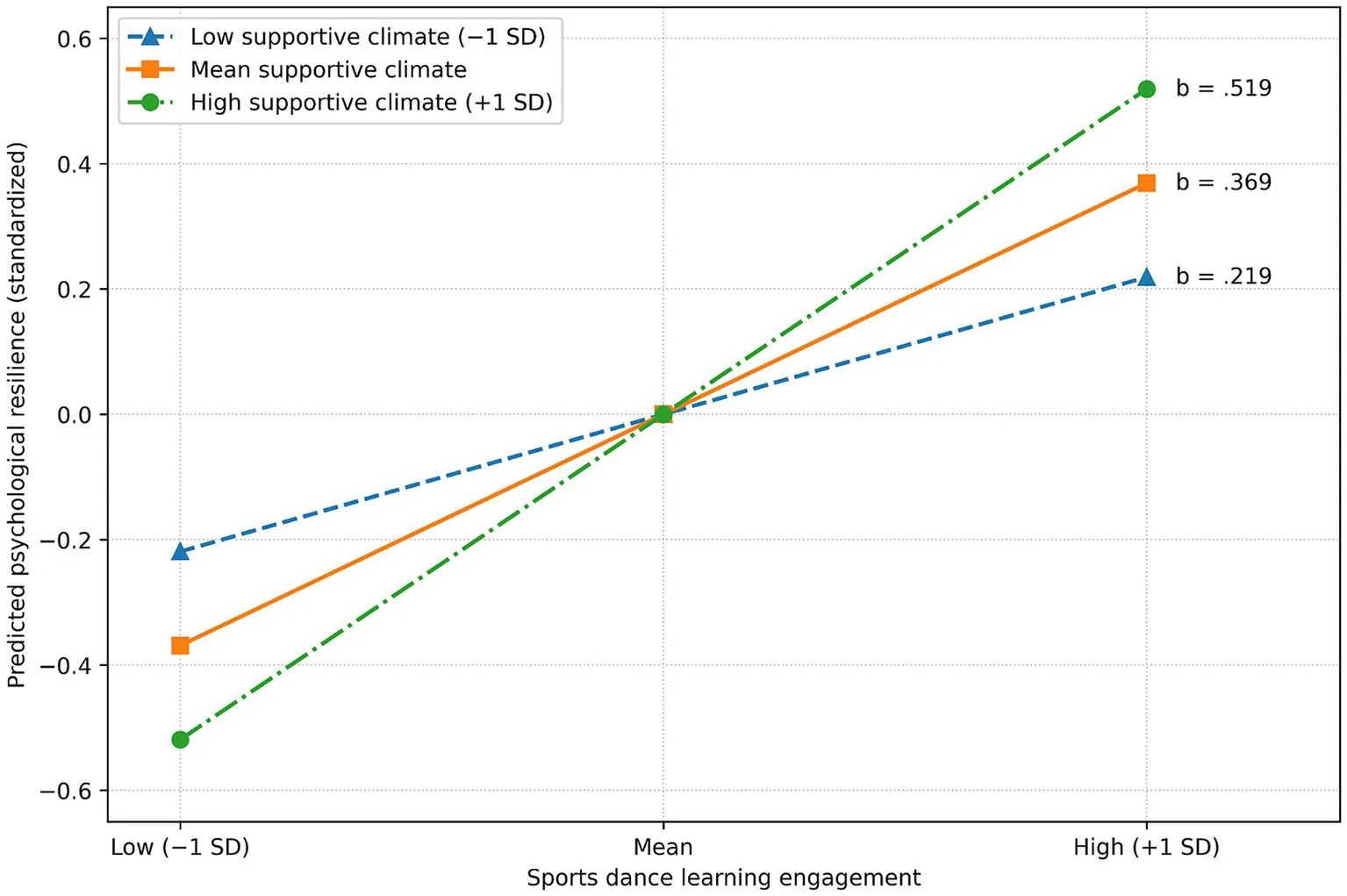
Interaction between sports dance learning engagement and supportive climate in relation to psychological resilience.

To further interpret the interaction, simple slopes for the association between sports dance learning engagement and psychological resilience were estimated at low (−1 SD), mean, and high (+1 SD) levels of supportive climate. The simple slope was 0.219 (95% CI [0.122, 0.316]) at low supportive climate, 0.369 (95% CI [0.294, 0.443]) at the mean level, and 0.519 (95% CI [0.414, 0.623]) at high supportive climate. The positive engagement–resilience association was statistically distinguishable from zero at all three levels of supportive climate and was stronger at higher levels of supportive climate. The interaction is illustrated in Figure 3.

The conditional indirect associations were subsequently examined using PROCESS Model 7. As shown in Table 8, the indirect association between sports dance learning engagement and SEC through psychological resilience was 0.077 at low supportive climate (95% CI [0.041, 0.120]), 0.130 at the mean level of supportive climate (95% CI [0.093, 0.172]), and 0.183 at high supportive climate (95% CI [0.133, 0.239]). All three bootstrap confidence intervals excluded zero, indicating statistically distinguishable indirect associations across the three examined levels of supportive climate.

**Table 8:** Bootstrap estimates of conditional indirect associations of sports dance learning engagement with SEC through psychological resilience.

Supportive climate	Indirect effect	SE	95% CI
Low (−1 SD)	0.077	0.020	[0.041, 0.120]
Mean	0.130	0.020	[0.093, 0.172]
High (+1 SD)	0.183	0.027	[0.133, 0.239]
Index of moderated mediation	0.053	0.014	[0.027, 0.080]

The index of moderated mediation was 0.053 (SE = 0.014, 95% CI [0.027, 0.080]). Because the bootstrap confidence interval excluded zero, the magnitude of the indirect association varied statistically across levels of supportive climate. Specifically, the indirect association was modestly stronger at higher levels of perceived supportive climate. Given the cross-sectional design, this pattern is interpreted as a conditional association rather than evidence that supportive climate causally strengthens a temporal mediation process.

Taken together, the results supported the hypothesized pattern of direct, indirect, and conditional associations. Sports dance learning engagement was positively associated with SEC both directly and indirectly through psychological resilience, and the magnitude of the indirect association varied across levels of supportive climate. Given the cross-sectional design, these findings represent statistical associations consistent with the hypothesized model rather than evidence of temporal or causal pathways.

## Discussion

The present study examined the associations among sports dance learning engagement, psychological resilience, supportive climate, and social–emotional competence (SEC) among Chinese university students. Overall, the findings were consistent with the proposed theoretical model: engagement was positively associated with both resilience and SEC, resilience was positively associated with SEC, and the indirect association through resilience varied across levels of supportive climate. These results should be interpreted as contemporaneous statistical relationships rather than evidence of temporal sequencing or causal mechanisms.

### Sports dance learning engagement and social–emotional competence

The positive association between sports dance learning engagement and SEC was consistent with the multidimensional engagement perspective proposed by Fredricks et al. (2004) and with social–emotional learning perspectives emphasizing self-regulatory and interpersonal functioning in educational contexts (Collaborative for Academic, Social, and Emotional Learning (CASEL), 2020; Durlak et al., 2011). Students who reported stronger behavioral participation, emotional involvement, and cognitive investment in sports dance learning also tended to report higher levels of self-awareness, self-management, relationship skills, and responsible decision-making. Sports dance provides a plausible setting for observing this relationship because learning commonly involves movement, partner coordination, feedback, emotional expression, and socially visible performance. These features make emotional regulation, communication, cooperation, and adaptive responding salient during learning. Dance and embodied-learning research has similarly linked movement-based participation with social interaction, emotional expression, and psychosocial functioning (Borowski, 2023; Hayashi et al., 2022; Zafeiroudi, 2023). However, the present study neither measured embodiment as an independent construct nor compared sports dance with other physical, artistic, or collaborative learning activities. The findings therefore do not show that sports dance has unique or stronger social–emotional effects than other educational contexts; rather, they indicate that established engagement–SEC relationships are observable within this particular movement-based and socially interactive setting.

The magnitude of the engagement–SEC association also suggests that engagement represents only one correlate of students’ social–emotional functioning. Other personal, instructional, interpersonal, and contextual characteristics are likely to be relevant. Reverse or reciprocal relationships are also plausible. Students with stronger SEC may, for example, be more comfortable with partner coordination, feedback, emotional expression, or public performance and may consequently become more engaged in sports dance learning. Longitudinal research is therefore needed to clarify whether engagement precedes changes in SEC, SEC predicts subsequent engagement, or the relationship develops reciprocally over time.

### Psychological resilience as an intermediate construct

The findings supported a statistically significant indirect association between sports dance learning engagement and SEC through psychological resilience. Students reporting greater engagement tended to report greater resilience, and greater resilience was in turn associated with higher SEC. This pattern is consistent with the theoretical ordering informed by Broaden-and-Build Theory (Fredrickson, 2001). Sports dance learning can involve repeated practice demands, correction, performance pressure, mistakes, physical fatigue, and interpersonal coordination, while greater engagement may also coincide with mastery experiences, enjoyment, social connectedness, and sustained involvement. These experiences are theoretically relevant to adaptive psychological functioning. Given the cross-sectional design, however, psychological resilience is more appropriately interpreted as an intermediate construct within the observed covariance structure than as a demonstrated developmental mediator.

The positive association between resilience and SEC requires particular attention because the constructs are conceptually adjacent. Resilience concerns adaptation and recovery in response to adversity or setbacks (Connor and Davidson, 2003; Masten, 2001), whereas SEC in the present study represents a broader set of CASEL-aligned competencies involving self-awareness, self-management, relationship skills, and responsible decision-making. Self-management is especially close conceptually to resilience, but the constructs were not empirically redundant. The measurement-model and discriminant-validity results supported empirical distinction between the resilience indicators and the dimensions used to represent SEC, although conceptual proximity between resilience and self-management should still be acknowledged. The statistically significant direct engagement–SEC association alongside the indirect association also indicates that resilience does not provide a complete statistical account of their relationship. Other variables, such as self-efficacy, perceived competence, belongingness, emotional intelligence, motivational regulation, or instructional and peer factors, may also contribute and should be examined in future longitudinal research.

### The contextual role of supportive climate

Supportive climate was associated with variation in the strength of the engagement–resilience relationship. The association was stronger at higher levels of perceived supportive climate, which is consistent with Conservation of Resources Theory and its emphasis on the relevance of contextual resources to personal adaptive functioning (Hobfoll, 1989). In sports dance learning, teacher encouragement, peer support, and psychological safety may be particularly relevant when students encounter correction, mistakes, interpersonal coordination demands, and socially visible performance. The simple-slope results showed that the engagement–resilience association remained statistically distinguishable from zero at low, mean, and high levels of supportive climate but increased progressively across these levels. Importantly, the interaction effect was modest (b = 0.150). Supportive climate should therefore be understood as one contextual correlate of the engagement–resilience association rather than as a dominant condition determining resilience.

The conditional indirect results followed the same pattern: the indirect association between engagement and SEC through resilience was larger at higher levels of supportive climate, and the index of moderated mediation was statistically distinguishable from zero. This finding is consistent with the proposed first-stage moderation model and supports a person-by-context interpretation in which students’ involvement and perceived contextual resources are jointly related to adaptive psychological functioning. It does not, however, demonstrate that changing supportive climate would causally increase resilience or SEC. Other contextual and individual factors, including instructional quality, prior experience, peer cohesion, perceived competence, and personality, may also contribute to the observed variation.

### Theoretical implications

The main theoretical contribution of the study lies in contextual integration and construct positioning rather than the identification of a new causal mechanism specific to sports dance. The findings show that relationships commonly discussed in educational psychology among engagement, adaptive psychological functioning, and SEC are observable within university sports dance education. This extends the contextual scope of engagement research to a movement-based, partner-coordinated, and performance-oriented setting without assuming that sports dance is theoretically unique or superior to other learning contexts. The study also clarifies the position of psychological resilience within the engagement–SEC model by treating resilience as an adversity-oriented adaptive capacity that is theoretically related to, but distinct from, broader social–emotional competencies. The discriminant-validity evidence supports this distinction, while the indirect association indicates that resilience occupies a meaningful intermediate position in the observed covariance structure.

The findings further provide a contextual interpretation of the engagement–resilience relationship. Supportive climate was associated with modest but statistically distinguishable variation in this relationship, consistent with the proposition that personal involvement and perceived contextual resources are jointly relevant to adaptive psychological functioning. The contribution is therefore not that supportive climate was shown to causally amplify resilience development, but that the engagement–resilience association differed systematically across students’ perceptions of their learning environments. Taken together, the study offers an integrative, context-specific application of established educational-psychology perspectives rather than evidence for a unique developmental mechanism inherent to sports dance.

### Practical implications

The findings offer tentative implications for sports dance instruction. Instructors and curriculum designers may consider learning environments that encourage active participation, constructive feedback, partner cooperation, and psychological safety, given that engagement, resilience, and supportive climate were positively associated with students’ reported social–emotional functioning. Learning activities that provide structured opportunities to respond to mistakes, performance pressure, and interpersonal coordination demands may also be relevant to students’ adaptive experiences. These implications should remain cautious because the study did not manipulate instructional practices, supportive climate, resilience, or SEC and therefore cannot establish that implementing particular strategies will improve these outcomes. The findings are better viewed as hypothesis-generating evidence that can inform future experimental or quasi-experimental studies examining specific instructional practices and changes in resilience and SEC over time.

### Limitations and future directions

Several limitations should guide interpretation of the findings. Most importantly, all focal variables were assessed concurrently. The indirect and conditional indirect effects therefore represent statistical decompositions of cross-sectional associations rather than evidence of temporal mediation, and reverse or reciprocal relationships remain plausible. The exclusive reliance on student self-report also creates potential common method concerns. Although Harman’s single-factor test, full-collinearity diagnostics, and CFA-based model comparisons did not support a simple single-factor explanation of the covariance structure, these procedures cannot eliminate common method variance. Social desirability, response style, shared measurement context, and general positive self-perceptions may have contributed to the observed associations. Future studies should employ longitudinal, cross-lagged, or intervention-based designs and combine student reports with teacher ratings, peer assessments, behavioral observations, or performance-based indicators.

The sampling and analytic structure also constrain inference. Participants were drawn from sports dance courses at three Chinese universities, limiting generalizability beyond comparable higher-education settings. In addition, class- and institution-level identifiers were not retained in the final analytic dataset, so potential clustering could not be modeled directly. Self-selection may also have influenced the findings because students who enroll in, remain in, or engage more strongly with sports dance may differ systematically in motivation, prior dance experience, physical confidence, interpersonal orientation, emotional regulation, or related characteristics. Future studies should retain class- and institution-level identifiers, assess relevant baseline characteristics, and, where feasible, employ multilevel analysis, comparison groups, or statistical approaches designed to reduce selection bias.

Finally, although sports dance was discussed as an embodied, partner-coordinated, and socially evaluative learning context, embodiment itself was not directly measured. The present findings therefore cannot establish that embodied learning processes explain the observed associations. Future studies could directly assess embodied engagement, interpersonal synchrony, movement-based emotional expression, or performance-related social evaluation and compare sports dance with other physical, artistic, and classroom-based activities. Such designs would allow stronger conclusions about which features are specific to sports dance and which reflect broader educational processes.

## Conclusion

This study examined how sports dance learning engagement, psychological resilience, supportive climate, and social–emotional competence are interconnected among university students in China. The findings indicated that engagement was positively associated with social–emotional competence both directly and indirectly through psychological resilience, and that the magnitude of this indirect association varied across levels of perceived supportive climate. Beyond the individual associations among the study variables, the results highlight the relevance of considering both personal and contextual resources when understanding students’ social–emotional functioning. By integrating engagement, resilience, supportive climate, and social–emotional competence within a single framework, the study provides a more contextualized understanding of how these constructs are related within an embodied, partner-coordinated, and socially evaluative learning setting. Rather than demonstrating a unique developmental mechanism specific to sports dance, the findings provide a context-specific test of established educational-psychology relationships. Because the study relied on cross-sectional self-report data, the observed direct, indirect, and conditional associations should not be interpreted as evidence of temporal sequencing or causal effects. Future longitudinal, multi-source, and intervention-based research is needed to examine the temporal ordering and potential longitudinal relationships among engagement, resilience, supportive climate, and social–emotional competence.

## Data Availability

The raw data supporting the conclusions of this article will be made available by the authors, without undue reservation.
